# Breath metabolomics for diagnosis of acute respiratory distress syndrome

**DOI:** 10.1186/s13054-024-04882-7

**Published:** 2024-03-23

**Authors:** Shiqi Zhang, Laura A. Hagens, Nanon F. L. Heijnen, Marry R. Smit, Paul Brinkman, Dominic Fenn, Tom van der Poll, Marcus J. Schultz, Dennis C. J. J. Bergmans, Ronny M. Schnabel, Lieuwe D. J. Bos, for the DARTS Consortium, Lieuwe D. J. Bos, Laura A. Hagens, Marcus J. Schultz, Marry R. Smit, Dennis C. J. J. Bergmans, Nanon F. L. Heijnen, Ronny M. Schnabel, Inge Geven, Tamara M. E. Nijsen, Alwin R. M. Verschueren

**Affiliations:** 1 Amsterdam UMC, Location AMC, Department of Intensive Care, University of Amsterdam, Meibergdreef 9, Room G3-228, 1105 AZ Amsterdam, The Netherlands; 2 Amsterdam UMC, Location AMC, University of Amsterdam, Pulmonary Medicine, Amsterdam, The Netherlands; 3 Amsterdam UMC, Location AMC, Division of Infectious Diseases, University of Amsterdam, Amsterdam, The Netherlands; 4 Amsterdam UMC, Location AMC, Center of Experimental and Molecular Medicine (CEMM), University of Amsterdam, Amsterdam, The Netherlands; 5 Department of Intensive Care, Maastricht University Medical Centre+, Maastricht, The Netherlands; 6 Maastricht University Medical Centre+, School of Nutrition and Translational Research in Metabolism (NUTRIM), Maastricht, The Netherlands; 7 Mahidol–Oxford Tropical Medicine Research Unit (MORU), Mahidol University, Bangkok, Thailand; 8 Nuffield Department of Medicine, University of Oxford, Oxford, UK

**Keywords:** Breath analysis, VOCs, ARDS, Prediction model

## Abstract

**Background:**

Acute respiratory distress syndrome (ARDS) poses challenges in early identification. Exhaled breath contains metabolites reflective of pulmonary inflammation.

**Aim:**

To evaluate the diagnostic accuracy of breath metabolites for ARDS in invasively ventilated intensive care unit (ICU) patients.

**Methods:**

This two-center observational study included critically ill patients receiving invasive ventilation. Gas chromatography and mass spectrometry (GC–MS) was used to quantify the exhaled metabolites. The Berlin definition of ARDS was assessed by three experts to categorize all patients into “certain ARDS”, “certain no ARDS” and “uncertain ARDS” groups. The patients with “certain” labels from one hospital formed the derivation cohort used to train a classifier built based on the five most significant breath metabolites. The diagnostic accuracy of the classifier was assessed in all patients from the second hospital and combined with the lung injury prediction score (LIPS).

**Results:**

A total of 499 patients were included in this study. Three hundred fifty-seven patients were included in the derivation cohort (60 with certain ARDS; 17%), and 142 patients in the validation cohort (47 with certain ARDS; 33%). The metabolites 1-methylpyrrole, 1,3,5-trifluorobenzene, methoxyacetic acid, 2-methylfuran and 2-methyl-1-propanol were included in the classifier. The classifier had an area under the receiver operating characteristics curve (AUROCC) of 0.71 (CI 0.63–0.78) in the derivation cohort and 0.63 (CI 0.52–0.74) in the validation cohort. Combining the breath test with the LIPS does not significantly enhance the diagnostic performance.

**Conclusion:**

An exhaled breath metabolomics-based classifier has moderate diagnostic accuracy for ARDS but was not sufficiently accurate for clinical use, even after combination with a clinical prediction score.

**Supplementary Information:**

The online version contains supplementary material available at 10.1186/s13054-024-04882-7.

## Introduction

Acute respiratory distress syndrome (ARDS) is a life-threatening condition that causes acute hypoxemic respiratory failure due to exudative pulmonary edema [1]. Prevalence of ARDS in the intensive care unit (ICU) is around 10%, and hospital mortality ranges from 35 to 45% [2]. Diagnosis of ARDS is based on timing, severity of hypoxemia, and presence of bilateral pulmonary infiltrates, that is not fully explained by cardiac dysfunction or fluid overload [3]. Thus, assessment of ARDS is based on clinical criteria with considerable inter-observer heterogeneity resulting in subjectivity [4]. An alternative approach is needed.

Biological markers can provide objective evidence for the pathophysiological processes of various diseases and may shed light into the injurious processes leading to alveolar injury [1]. Biomarkers of alveolar injury have shown reasonable diagnostic accuracy for ARDS [5, 6]. However, these biomarkers are typically measured in plasma while the pathological processes of ARDS mainly occurs locally in the lung. As such, plasma biomarkers may poorly reflect alveolar processes and assessing biomarkers directly from the lung may give a better indication [7].

Hundreds to thousands of volatile organic compounds (VOCs) can be identified in exhaled breath using gas chromatography–mass spectrometry (GC–MS). In a recent systematic review, a panel of VOC metabolites was identified as a promising method for ARDS diagnosis, showing high diagnostic accuracy and low bias [8]. Indeed, VOCs in exhaled breath offer a direct and real-time reflection of the comprehensive volatile metabolic profile within the lungs. The non-invasive nature and bedside accessibility of VOC capture enhance their potential to serve as the perfect biomarker, concurrently enabling early detection and dynamic monitoring capabilities. In recent years, numerous studies have delved into the diagnostic accuracy of those non-invasive markers in the detection of various respiratory diseases [9–13]. Among these, breath benzaldehyde, octane and 3-methylheptane were previously identified as promising biomarkers for ARDS, yet their diagnostic accuracy did not withstand external validation [14–16].

In the present study, we aimed to evaluate the diagnostic accuracy of a novel exhaled breath metabolomics-based classifier. We hypothesized that a data-driven classifier can accurately diagnose ARDS, also after external validation. Considering the lack of a gold standard for ARDS diagnosis, we used the combined judgment of an expert panel as reference standard. Secondly, we hypothesized that diagnostic accuracy improved when the classifier was combined with the lung injury prediction score (LIPS), a clinical classifier. Finally, we explored the relationship between VOCs in exhaled breath and plasma biomarkers in an attempt to elucidate a possible biochemical origin of the VOCs we found in exhaled breath.

## Methods

### Study design and ethical consideration

This was a pre-planned secondary analysis of the DARTS project, a prospective multicenter observational cohort study aimed to evaluate the diagnostic accuracy of several imaging and biomarker tests for ARDS. This study used data from consecutive patients admitted to the ICUs of the Amsterdam UMC, location AMC and the Maastricht University Medical Center+ (MUMC +), two university hospitals in the Netherlands [17]. The study enrollment continued for two years, from March 26, 2019, to March 1, 2021. The Institutional Review Board of both centers waived the need for ethical approval of the protocol (W18_311#18.358 and 2019–1137). The study was registered with the “DARTS study” tag at the Dutch trial register (NL8226, www.trialregister.nl). Written informed consent was obtained from all patients or their relatives.

### Patient recruitment

Consecutive adult patients with an expected duration of invasive ventilation of at least 24 h were recruited as soon as possible, but not later than 48 h, after starting invasive ventilation in the ICU. The exclusion criteria were: (1) receiving 48 h of invasive ventilation during the seven days before inclusion, (2) tracheostomy, (3) life expectancy less than 24 h, (4) lack of written informed consent.

### Reference standard: ARDS diagnosis

ARDS was defined using the Berlin criteria [3]. To limit the influence of inter-observer variation on the ARDS labeling in this study, three experts scored the presence of ARDS independently based on a review of each clinical case, which included data on comorbidities, ventilator and gas-exchange parameters and available chest imaging [18]. Based on the available data, each expert scored their likelihood of ARDS from 1 (certain no ARDS) to 8 (certain ARDS). By summarizing the scores, patients were categorized with a “certain” label when the experts agreed and with an “uncertain” label when there was disagreement or when the scores were equivocal. The patients with uncertain ARDS diagnosis were discussed in a consensus meeting to be classified into either “likely ARDS” or “likely no ARDS”. Assessment of the reference test was blinded for any results from the index test. The process of classification can be found in the supplement material (Description of ARDS classification procedure and Additional file 1: Table S1).

### Index test: exhaled breath analysis

As previously described in the DARTS protocol [17], the exhaled breath samples were collected by a breath gas sampler through a side-stream connection of a polytetrafluoroethylene (PTFE) tube on the expiratory limb after the heat and moisture exchange (HME) filter with a flow of 200 mL/min, and the collection lasted for 6 mins. In this way, VOCs in exhaled breath were absorbed onto the sorbent tubes and the tubes were stored and subsequently analyzed within 2 weeks. During the first 48 h of invasive mechanical ventilation, a first breath sample was drawn, followed by a second sample on the following day. The sorbent tubes were heated to 250 degrees Celsius (Markes, TD100), focused on a cold trap and rapidly injected onto an Inertcap 5MS/Sil GC column with a flow of 1.2 mL/min. In the mass-spectrometer, compounds were fragmented using electron ionization and a quadrupole mass spectrometer detected the fragment ions. The base peak of each VOC constituted a new variable and was used as independent predictor variable in the subsequent analyses [19]. Patients whose breath sample analyses were unsuccessful due to technical malfunctions in the GCMS machine, rendering it inoperable for the analyses, were excluded from the study.

### Additional biomarker testing

Blood samples were collected for plasma biomarker analysis. Biomarkers were measured using a Luminex multiplex assay (R&D systems, Abington, UK) and Bioplex 200 (Bio-Rad, Hercules, California, USA) according to the manufacturer’s protocols.

### Sample size justification

This was a secondary analysis of the DARTS project, which included more than 500 patients to assess ARDS diagnosis. To ensure sufficient statistical power of this analysis, the sample size of the patients with certain diagnosis in the derivation cohort was calculated retrospectively using the *pmsampsize* package in Rstudio (version 4.0.3) [20, 21]. With an expected C-statistic (AUROCC) of 0.90 and a prevalence of ARDS of 10.4% in ICU, the sample size should reach 179 for a model when we choose five independent variables as prediction parameters [2]. The sample size of the validation cohort was given based on the number of inclusions in the Maastricht hospital.

### Statistical analysis

Patients recruited in AMC with certain labels were allocated to the derivation cohort to develop a classifier (called “VOC-ARDS score”), while patients with “certain” labels who were recruited in Maastricht acted as the validation cohort to evaluate its performance. The two datasets were kept independent of each other.

To select the best predictors, a random forest model with the log10 transformed ion count of the base peak of all VOCs as predictive variables was trained in the derivation cohort (*caret* package) [22]. Combining the results of variable importance ranking both in mean decrease in Gini plot and mean decrease in accuracy plot (*randomForest* package) [23], the top five most important VOCs were identified. The selected VOCs were included as independent variables in a logistic regression model. The VOC-ARDS score was derived by the sum of selected variables multiplied by their corresponding coefficient.

The discriminative performance of this model was evaluated using the area under the receiver operating characteristic curve (AUROCC) with 95% confidence intervals. As newly derived multivariable diagnostic scores are prone to overfitting, we tested the diagnostic accuracy of the VOC-ARDS score in the validation cohort. To get a reliable estimate of accuracy, we included all patients, also when the three experts had conflicting or uncertain classifications for ARDS. As a sensitivity analysis, this comparison was repeated for the second sample to assess the stability of the discrimination over time. In order to integrate biological data with the clinical patients’ risk, the lung injury prediction score (LIPS) was added as a covariate to the logistic regression and improvements in the diagnostic accuracy were evaluated, the difference between AUROCCs were compared using *roc.test()* function with “bootstrap” method using the *pROC* package [24]. Linear regression was used to quantify the association between the selected VOCs with plasma biomarkers that had previously been linked to the pathophysiological processes involved in ARDS development. To identify patterns in the data, the correlation coefficients between VOCs and plasma biomarkers were visualized in a heatmap.

Data are expressed as median (interquartile range) for continuous variables and number (%) for categorical variables. Differences between groups were tested by the Mann–Whitney *U* test or the Fisher’s exact test, as appropriate. A *p* value < 0.05 was considered statistically significant. All data analysis was performed in R studio (version 4.0.3).

## Results

### Included patients

**Fig. 1:**
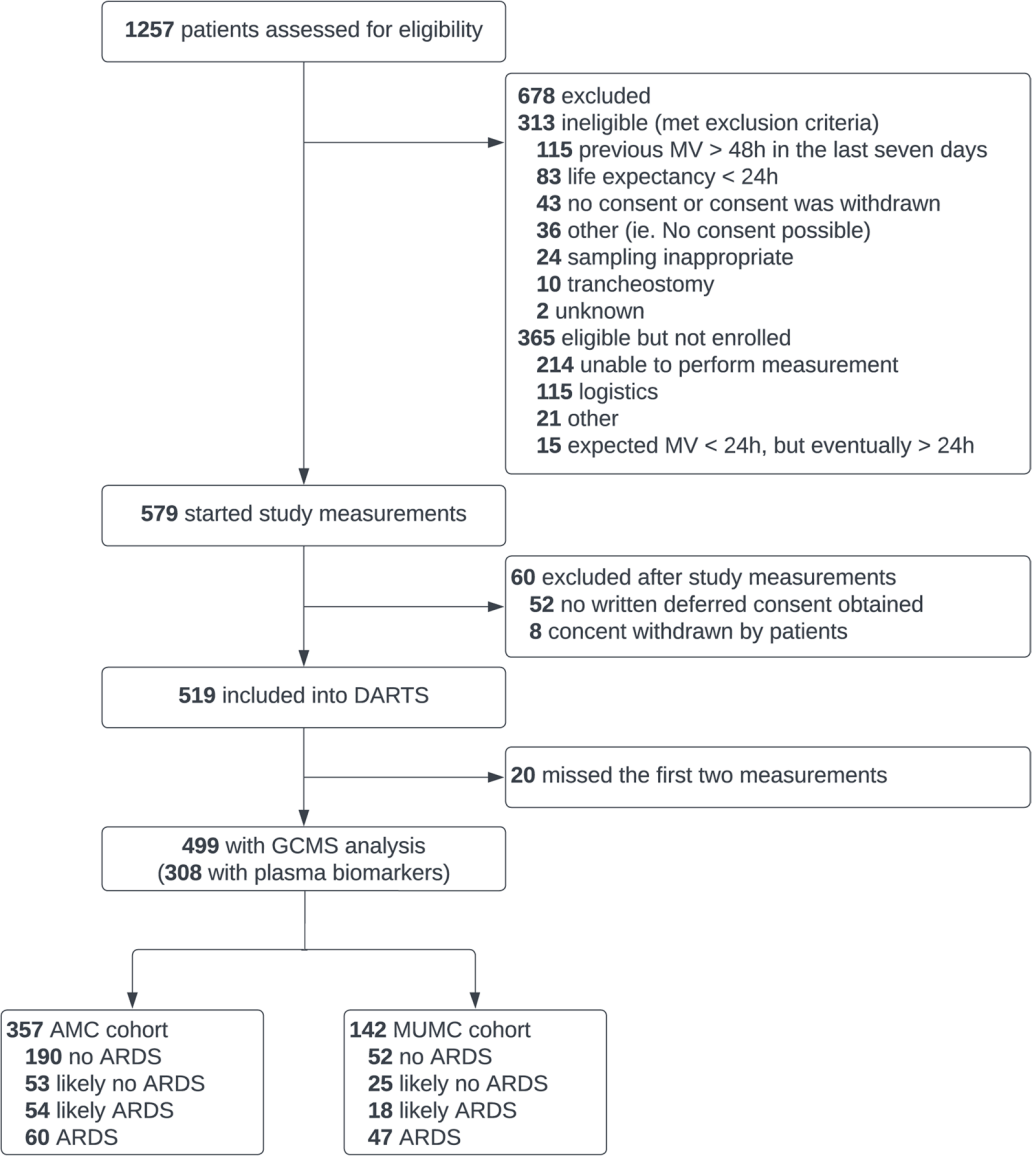
Flowchart of screening and inclusion process. *MV* Mechanical ventilation, *GCMS* Gas chromatography-mass spectrometry, *ARDS* Acute respiratory distress syndrome

A total of 499 patients were included in this study, of whom 357 patients were recruited from AMC (AMC cohort) and 142 patients were from MUMC + (MUMC cohort). Sixty (17%) patients recruited in AMC and 47 patients (33%) recruited in MUMC + were classified as having certain ARDS (Fig. 1). A total of 250 patients in AMC cohort had certain labels and were used for model derivation. From all the included patients, the diagnosis of ARDS remained uncertain in one third of them, and approximately half of these patients were finally classified as likely having ARDS in a consensus meeting (Fig. 1). Patient characteristics and ventilation parameters of the patient cohort used to derive and validate the model are presented in Table 1 and supplemental Additional file 1: Table S2, respectively.

**Table 1:** Characteristics of the patient cohort used to derive the VOC-ARDS score

	Certain ARDS labels in AMC		
	ARDS (*n* = 60)	Non-ARDS (*n* = 190)	*P* value
*Patients characteristics*			
Age, years mean (SD)	60.2 (13.8)	61.8 (16.1)	0.47
Male, *n* (%)	45 (75)	130 (68)	0.419
Smoker, *n* (%)	48 (80)	146 (77)	0.738
BMI, kg/m2 median [IQR]	26.9 [23.7, 30.7]	25.8 [23.0, 30.0]	0.191
*Admission characteristics*			
Admission type, *n* (%)			0.001
Emergency surgical	2 (3.3)	37 (19.5)	
Medical	54 (90.0)	123 (64.7)	
Planned surgical	4 (6.7)	30 (15.8)	
*Admission condition, n* (%)			
Trauma	2 (3.3)	34 (17.9)	0.01
Neurosurgery	2 (3.3)	39 (20.5)	0.003
Shock	1 (1.7)	15 (7.9)	0.157
Extrapulmonary sepsis	3 (5.0)	31 (16.3)	0.044
Pancreatitis	0 (0.0)	3 (1.6)	0.765
*Comorbidities, n* (%)			
Diabetes	13 (21.7)	35 (18.4)	0.713
Active malignancy	11 (18.3)	22 (11.6)	0.259
Immunocompromised	5 (8.3)	10 (5.3)	0.575
*Cause of ARDS, n* (%)			< 0.001
Non pulmonary	4 (6.7)	–	
Pulmonary	56 (93.3)	–	
*ARDS severity, n* (%)			< 0.001
Mild	3 (5.0)	–	
Moderate	30 (50.0)	–	
Severe	27 (45.0)	–	
Pneumonia, *n* (%)	54 (90.0)	28 (14.7)	< 0.001
Covid-19, *n* (%)	29 (48.3)	2 (1.1)	< 0.001
Apache II score, median [IQR]	20 [15, 22]	21 [15, 26]	0.023
SOFA score, median [IQR]	8 [5, 12]	10 [8, 12]	0.011
LIPS, median [IQR]	6 [6, 8]	5 [3, 6]	< 0.001
Pre-ICU LOS, days median [IQR]	2 [0, 5]	1 [0, 3]	0.227
*Mechanical ventilation and gas exchange, median [IQR]*			
P max, cmH_2_O	23 [18, 28]	18 [14, 22]	< 0.001
PEEP, cmH_2_O	10 [8, 12]	5 [5, 8]	< 0.001
Driving pressure, cmH_2_O	12 [8, 17]	12 [8, 15]	0.458
V_T_, mL	518 [386, 624]	470 [402, 532]	0.162
V_T_/PBW, ml/kg	7.8 [6.3, 9.3]	7.1 [6.2, 8.6]	0.068
Compliance, mL/cmH_2_O	34.5 [24.1, 51.5]	36.8 [27.6, 57.8]	0.173
RR, breaths/min	21 [16, 28]	18 [15, 23]	0.006
PaO_2_/FiO_2_, mmHg†	105 [81, 142]	263 [169, 336]	< 0.001
Duration MV, hours	20 [10, 32]	22 [12, 32]	0.671
Outcomes			
ICU LOS, days median [IQR]	9 [6, 19]	6 [3, 11]	< 0.001
Hospital LOS, days median [IQR]	19 [10, 31]	16 [8, 31]	0.394
ICU mortality, *n* (%)	25 (41.7)	60 (31.6)	0.2
30d mortality, *n* (%)	25 (41.7)	70 (36.8)	0.604
90d mortality, *n* (%)	27 (45.0)	74 (38.9)	0.495
1 year mortality, *n* (%)	27 (45.0)	76 (40.0)	0.592

Data are presented as *n* (%), median [IQR] or mean (SD). *P* values were calculated using Chi-square, *T* test or Mann–Whitney *U* test depending on the type and distribution of the variable. PBW is calculated as: PBW male = 50 + 0·91 * (cm of height—152·4) and PBW female = 45·5 + 0·91 * (cm of height—152·4)

^†^PF ratio worst measured in the 24 h before sampling

*ARDS* Acute respiratory distress syndrome, *BMI* Body mass index, *APACHE II* Acute physiology and chronic health evaluation II, *SOFA* Sequential organ failure assessment, *LIPS* Lung injury prediction score, *ICU* Intensive care unit, *LOS* Length of stay, *PaO*_*2*_ Partial pressure of oxygen, *FiO*_*2*_ Fraction of inspired oxygen, *MV* Mechanical ventilation, *PEEP* Positive end-expiratory pressure, *RR* Respiratory rate. *Vt* Tidal volume, *PBW* Predicted body weight

### Development of the classifier

The random forest variable importance analysis resulted in the selection of five VOCs: 1-methylpyrrole [Chemical Abstracts Service (CAS): 96–54-8], 1,3,5-trifluorobenzene (CAS: 372–38-3), methoxyacetic acid (CAS: 625–45-6), 2-methylfuran (CAS: 534–22-5), 2-methyl-1-propanol (CAS: 78–83-1; Additional file 1: Figure S1) and those were included in a multivariable logistic regression model. The coefficients of these parameters and the diagnostic prediction model derived VOC-ARDS score are presented in supplemental Additional file 1: Table S3. The relative abundance of the selected five VOCs in patients with different diagnostic classifications is also presented (Additional file 1: Figure S2).

### Diagnostic accuracy of VOC-ARDS score

**Fig. 2:**
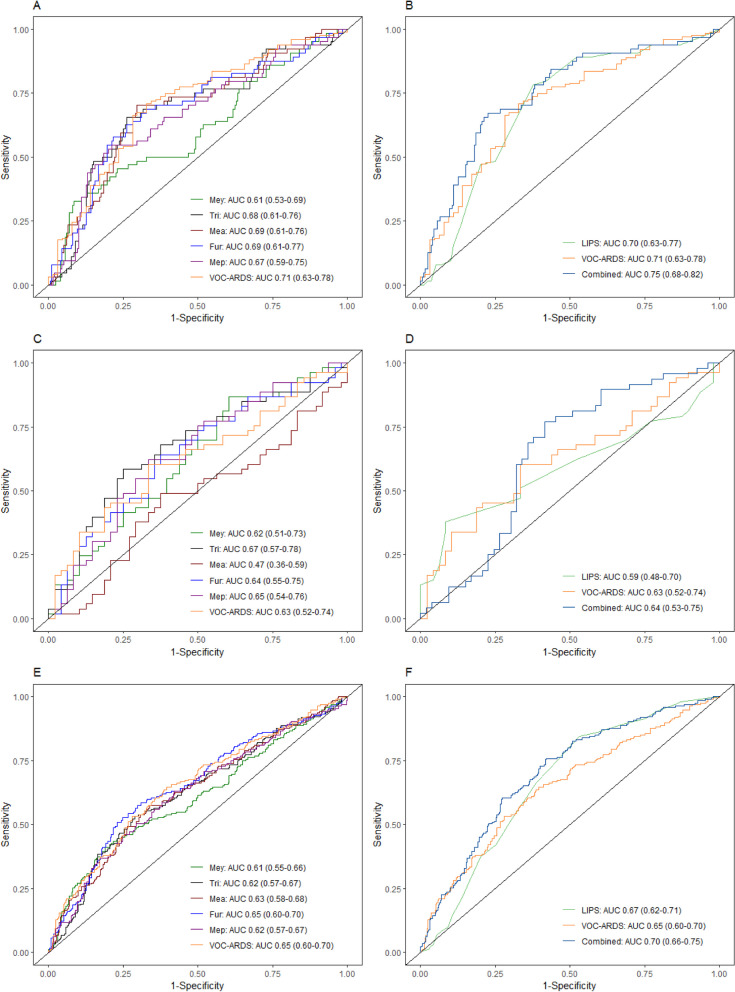
Receiver operating characteristics (ROC) curve per metabolite that were included in the VOC-ARDS score classifier and the combined VOC-ARDS classifier with the lung injury prediction score (LIPS) for each cohort. **A**–**B** in the derivation cohort. **C–D** in the validation cohort. **E**–**F**: in all the included patients. *Mey* 1-methylpyrrole, *Tri* 1,3,5-trifluorobenzene, *Mea* Methoxyacetic acid, *Fur* 2-methylfuran, *Mep* 2-methyl-1-propanol, *AUC* Area under curve, *VOCs* Volatile organic compounds, *LIPS* Lung injury prediction score

In the derivation cohort, which included patients with certain ARDS labels only, the diagnostic accuracy of VOC-ARDS score was acceptable (AUROCC of 0.71, CI 0.63–0.78; Fig. 2A). When patients who had uncertain labels based on conflicting evaluations by the expert panel were also considered, the VOC-ARDS score showed an AUROCC of 0.67 (CI 0.61–0.73; Additional file 1: Figure S3 and Table S4).

To validate the diagnostic performance of VOC-ARDS score in a new population, we applied the identical logistical regression model on the validation cohort. The diagnostic accuracy was lower (AUROCC of 0.63, CI 0.52–0.74; Fig. 2C), and the diagnostic accuracy decreased further when patients in MUMC cohort with uncertain labels were also considered (AUROCC of 0.58 CI 0.48–0.67; Additional file 1: Figure S3 and Table S5). Calibration of the VOC-ARDS score is shown in Additional file 1: Figure S4. In a sensitivity analysis using the second breath sample to test the diagnostic accuracy of the VOC-ARDS score, similar results were obtained (Additional file 1: Figure S5).

### Combination with LIPS

The diagnostic accuracy of the LIPS for ARDS had an AUROCC of 0.67 (CI 0.62–0.71), in a similar range as the VOC-ARDS score. The accuracy of the VOC-ARDS score was significantly but marginally increased from 0.65 (CI 0.60–0.70) to 0.70 (CI 0.66–0.75) when LIPS was added to the model (P < 0.01, Fig. 2E and F). Similar changes were seen when data were split for the derivation and validation cohort (Fig. 2B and D).

### Comparison with plasma biomarkers

**Fig. 3:**
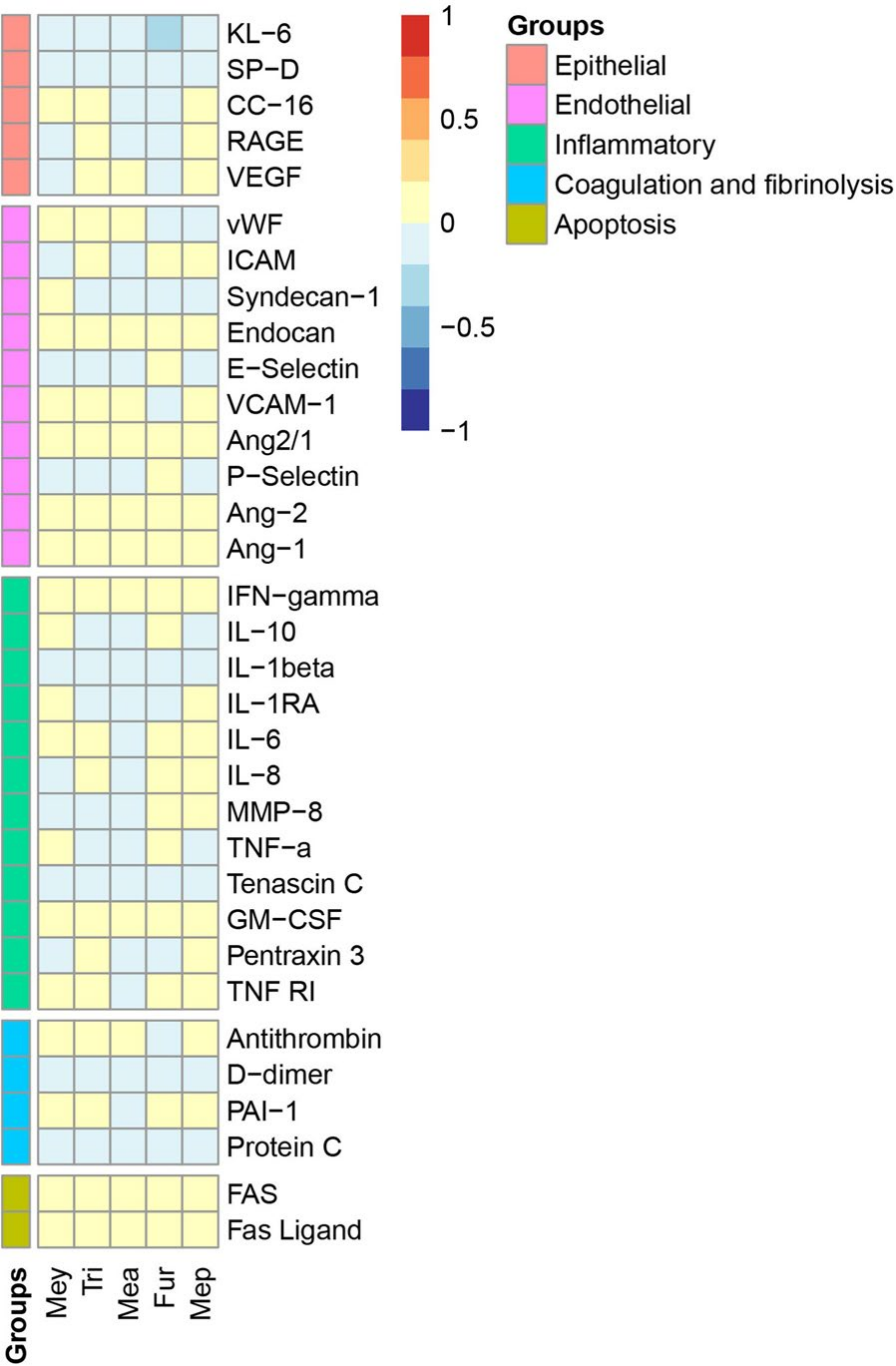
The association between exhaled breath VOCs and plasma biomarker levels. Every correlation coefficient between two variables was calculated using spearman correlation, color depth of each box indicates the correlation strength. *Mey* 1-methylpyrrole, *Tri* 1,3,5-trifluorobenzene, *Mea* Methoxyacetic acid, *Fur* 2-methylfuran, *Mep* 2-methyl-1-propanol. *Ang-1* Angiopoietin-1, *Ang-2* Angiopoietin-2, *Ang2/1* The ratio of Angiopoietin-1/ Angiopoietin-2, CC-16 = Club (Clara) cell protein 16, *VEGF* Vascular endothelial growth factor, *GM-CSF* Granulocyte–macrophage colony-stimulating factor, *ICAM* Intercellular adhesion molecule, *IFN* Interferon, *IL* Interleukin, *KL-6* Krebs von den Lungen-6, *MMP-8* Matrix metalloproteinase-8, *PAI-1* Plasminogen activator inhibitor-1, *RAGE* Receptor for advanced glycation end-products, *SP-D* Surfactant protein-D, *TNF* Tumor-necrosis factor, *TNF RI* Tumor-necrosis factor receptor 1, *VCAM* Vascular adhesion molecule, *VEGF* Vascular endothelial growth factor, *vWF* Von Willebrand Factor

Plasma samples from 308 patients (including 70 patients with certain ARDS, 148 certainly without ARDS and 90 patients with “uncertain” label) were collected and included in the biomarkers analysis (Additional file 1: Table S6). No strong significant correlation was found between the concentrations of the selected five VOCs and the measured plasma markers. The level of Krebs von den Lungen-6 (KL-6) in plasma was observed to have a weak negative correlation with the concentrations of the VOCs, correlation coefficients ranging from − 0.218 to − 0.103 (Fig. 3, Additional file 1: Table S7).

## Discussion

In this study, we derived and validated an exhaled breath metabolomics-based classifier to diagnose ARDS in a multicenter setting. The results of this study demonstrate that the exhaled breath patterns differ between patients with and without ARDS, but that the diagnostic accuracy of a model that captures these differences is insufficient for use in clinical practice. When combining the classifier with clinical information, in the form of the LIPS, the discriminating capacity slightly improved but remained insufficient. We observed a weak correlation between the selected breath metabolites and plasma biomarker of epithelial injury, suggesting that this process may contribute to the observed biological differences.

Several studies have evaluated the diagnostic potential of exhaled breath metabolomics for ARDS and have reported higher accuracies than those reported here [13, 25, 26]. However, this multicenter study with the largest sample size, utilizing GC–MS to identify exhaled metabolites, offers a superior design compared to previous research. Prior studies encountered limitations, including single-center setting and small patient cohorts, leading to challenges in replicating high diagnostic accuracy [15, 16, 26]. More recently, Heijnen et al. tested the diagnostic accuracy of octane and acetaldehyde in exhaled breath to identify ARDS in critically ill patients suspected of ventilator-associated pneumonia and also found a low diagnostic accuracy [13]. In line with these results, exhaled breath profiling with an electronic nose did provide low diagnostic accuracy [25]. Taken together, we should treat exhaled breath diagnostic tests for ARDS cautiously and await extensive independent validation in sufficient sample size before accepting a test.

The sufficient to good diagnostic accuracy observed in this study is lower than the accuracy of some other non-invasive diagnostic tests, such as plasma biomarkers and lung ultrasound [27–29]. A recent systematic review on the topic concluded that a higher risk of bias contributed to a higher observed diagnostic accuracy, as the median AUROCC was 0.84 for high-bias studies and 0.75 for the low-bias studies [8]. In the latter category, two plasma biomarkers (Club cell protein 16 and soluble receptor for advanced glycation end-products) and two panels of breath metabolites were found to discriminate ARDS patients with good accuracy [15, 26, 30, 31]. We could not validate the diagnostic accuracy of such breath tests here. Yet, the identified exhaled breath metabolites were weakly correlated with the biomarker of alveolar injury, suggesting that they could reflect changes in the compartment of interest.

In the present study, we identified five VOCs with decreased concentrations in ARDS patients: 1-methylpyrrole, 1,3,5-trifluorobenzene, methoxyacetic acid, 2-methylfuran, and 2-methyl-1-propanol. Most of these molecules cannot be attributed to the process of metabolism in human body. Instead, they have been more commonly associated with microbial metabolism such as the production of branched acetic acids by *Staphylococcus aureus* [32], a common pathogen found in the respiratory tract of ICU patients. Additionally, 2-methyl-1-propanol and 2-methylfuran are often reported as fungal metabolites in previous literatures [33–35], and the latter can also be produced by some bacterial strains [36]. The previous studies have suggested that the metabolite composition of COVID patient’s exhaled breath is different from that of healthy subjects [37, 38]. In our study, the proportion of COVID patients in ARDS group is significantly higher than in the control group, which could affect the breath profiles of the patients in ARDS group and thus be different from the control group. We are unsure about the metabolic origin of 1-methylpyrrole. Taken together, the identification of these particular VOCs as biomarkers for ARDS might reflect an association between the respiratory volatile metabolome and the microbiome, which is in line with previous studies on this topic [39, 40]. Additional factors may include exogenous sources. For example, 2-methylfuran is a constituent of cigarette smoke and has previously been found in the exhaled breath of smokers [41, 42]. Fluor compounds like trifluorobenzene are most likely from an industrial source and could be regarded as the contamination of breath.

All discriminative VOCs were found in lower concentrations in ARDS patients compared to non-ARDS patients. This contrasts to previous observations and the cause is yet unclear, although several hypotheses arise. First, the lung microbiome might be altered by host response mechanisms, changes in local metabolites due to pulmonary edema and the administration of drugs. Such alterations to the lung microbiome composition might explain differences in exhaled VOC profiles, certainly considering the types of molecules identified in the current study. A second hypothesis is that the molecules originate from the systemic circulation and that decreased diffusion and ventilation-perfusion mismatch result in less exchange of these gasses, resulting in lower exhaled concentrations. This is evidenced in the current study by the negative correlation between the selected VOCs and plasma KL-6, a biomarker expressed on the surface of alveolar type II cells following epithelial cell damage and linked to diagnosis and prognosis of ARDS [6, 43]. As such, an elevated KL-6 likely represents increased alveolar injury and worsened gas exchange that might explain the reduced concentration of the selected VOCs observed in the present study. However, we were unable to draw definitive conclusions regarding the underlying mechanisms due to the lack of a strong association both between the exhaled VOCs and plasma biomarkers, as well as between the abundance of exhaled VOCs and ARDS diagnosis. A final, more skeptical theory would be that all observed associations can be attributed to false-discovery. Although the validation of the biomarker score in a validation cohort limits this possibility, we have previously experienced this problem when discovering and validating octane as a breath biomarker for ARDS [15, 16].

As discussed earlier, we attempted to limit all risks of bias that could have overestimated the diagnostic accuracy in this study. Yet, several potential limitations remained. First, there are inherent difficulties with the clinical diagnosis of ARDS and there is no ground truth. Patients did not receive histopathology examination of lung tissue due to the limitations of clinical practice and if they had, there would remain considerable discussion on how to classify ARDS [18]. However, we tried to avoid the influence of single observers in the diagnosis of ARDS by using multiple observers to gain consensus. Second, ARDS is considered to be a heterogeneous condition, and it may be impossible to capture one biological signal to uniformly classify patients who have different pathophysiological abnormalities [44]. Moreover, it is necessary to recognize the inherent technological constraints that are inevitably present during the sample processing. We sampled after the HME filter because high water vapor pressure in exhaled breath would result in loading of high concentrations of water on the sorbent medium and transfer it to the GC column resulting poor peak differentiation during gas chromatography, decay of the column and retention time shift that would limit the comparison of GC results over long periods of time required for the inclusion of a large number of patients. Although the VOCs in exhaled breath we were interested are not easily soluble in water and would not be captured by the water in the HME, we cannot exclude minor differences in our conclusions due to the selected sampling method. Additionally, the absence of positive findings in this study merely suggests that exhaled VOCs may not suffice as a reliable classifier for identifying ARDS patients. However, it is important to acknowledge the potential intriguing biological information within exhaled breath. Future investigations may consider exploring metabolites and proteins captured by the HME filter, which extend beyond the realm of VOCs. Furthermore, our GC–MS analysis captured 248 metabolites, and thus, does not encompass the entirety of metabolites present in nature, which could potentially result in the omission of certain meaningful metabolites that might contribute to the identification of ARDS. Finally, we only evaluated the performance of the breath test alone and in combination with a clinical prediction score (LIPS). We therefore cannot exclude that the identified markers have more diagnostic value when combined with other biological, physiological or imaging data.

The presented data imply that exhaled breath metabolomics is unsuitable as a stand-alone diagnostic test for ARDS. Due to the heterogeneous nature of the condition and the observed correlation between exhaled breath metabolites and the marker of alveolar injury, they might be better suited to the identification of ARDS subphenotypes. Before we can link the exhaled breath metabolites to subphenotypes, we need more in-depth knowledge on their biochemical origin and the pathophysiological processes they represent. Up to this point, we should be cautious interpreting results of exhaled breath analysis studies and in particular exhaled breath analysis for diagnosis in ARDS.

To conclude, an exhaled breath metabolomics-based classifier has a sufficient diagnostic accuracy for ARDS but is not good enough for use in clinical practice. Combining this classifier with a clinical prediction score slightly improved its diagnostic accuracy, yet it remained too low for clinical practice.

### Supplementary Information

**Additional file 1. Table S1.** Confidence scores for meeting the Berlin Definition. **Figure S1.** Va riable importance in the predictive model. **Figure S2.** Comparison of the relative abundance of VOCs in different groups based on classification of ARDS of both centers. **Figure S3.** Diagnostic accuracy of the VOC-ARDS score classifier per center. **Figure S4.** Calibration plot of the prediction model. **Figure S5.** Diagnostic accuracy in the samples from the second measurement. **Table S2.** Characteristics of the patient cohort used to validate the VOC-ARDS score. **Table S3.** Development of the VOC-ARDS score. **Table S4.** Patient characteristics for AMC cohort. **Table S5.** Patient characteristics for MUMC cohort. **Table S6.** Biomarkers with abbreviations. **Table S7.** Spearman correlation coefficients between plasma KL-6 and the selected five VOCs.

## Data Availability

Data and research materials used in this study are available upon request to qualified researchers for purposes of replication, further analysis, and academic collaboration. We are committed to promoting transparency and facilitating the sharing of resources to advance scientific knowledge. Please contact the corresponding author for inquiries regarding data and material access.

## References

[1] Meyer NJ, Gattinoni L, Calfee CS (2021). Acute respiratory distress syndrome. Lancet.

[2] Bellani G, Laffey JG, Pham T (2016). Epidemiology, patterns of care, and mortality for patients with acute respiratory distress syndrome in intensive care units in 50 countries. JAMA.

[3] Force ADT, Ranieri VM, Rubenfeld GD (2012). Acute respiratory distress syndrome: the Berlin definition. JAMA.

[4] Allardet-Servent J, Forel JM, Roch A (2009). FIO_2_ and acute respiratory distress syndrome definition during lung protective ventilation. Crit Care Med.

[5] Determann RM, Royakkers AA, Haitsma JJ, Zhang H, Slutsky AS, Ranieri VM, Schultz MJ (2010). Plasma levels of surfactant protein D and KL-6 for evaluation of lung injury in critically ill mechanically ventilated patients. BMC Pulm Med.

[6] Kondo T, Hattori N, Ishikawa N (2011). KL-6 concentration in pulmonary epithelial lining fluid is a useful prognostic indicator in patients with acute respiratory distress syndrome. Respir Res.

[7] Heijnen NFL, Hagens LA, Smit MR (2021). Biological subphenotypes of acute respiratory distress syndrome may not reflect differences in alveolar inflammation. Physiol Rep.

[8] Hagens LA, Heijnen NFL, Smit MR, Schultz MJ, Bergmans D, Schnabel RM, Bos LDJ (2021). Systematic review of diagnostic methods for acute respiratory distress syndrome. ERJ Open Res.

[9] Bos LD, Wang Y, Weda H (2014). A simple breath sampling method in intubated and mechanically ventilated critically ill patients. Respir Physiol Neurobiol.

[10] Żuchowska K, Filipiak W (2023). Modern approaches for detection of volatile organic compounds in metabolic studies focusing on pathogenic bacteria: current state of the art. J Pharmac Anal.

[11] Munoz-Lucas MA, Wagner-Struwing C, Jareno-Esteban J (2013). Differences in volatile organic compounds (VOC) determined in exhaled breath in two populations of lung cancer (LC): with and without COPD. Eur Respir J.

[12] Ibrahim W, Cordell RL, Wilde MJ (2021). Diagnosis of COVID-19 by exhaled breath analysis using gas chromatography-mass spectrometry. ERJ Open Res.

[13] Heijnen NFL, Hagens LA, van Schooten FJ (2022). Breath octane and acetaldehyde as markers for acute respiratory distress syndrome in invasively ventilated patients suspected to have ventilator-associated pneumonia. ERJ Open Res.

[14] Hagens LA, Verschueren ARM, Lammers A (2021). Development and validation of a point-of-care breath test for octane detection. Analyst.

[15] Bos LD, Weda H, Wang Y (2014). Exhaled breath metabolomics as a noninvasive diagnostic tool for acute respiratory distress syndrome. Eur Respir J.

[16] Hagens LA, Heijnen NFL, Smit MR (2023). Octane in exhaled breath to diagnose acute respiratory distress syndrome in invasively ventilated intensive care unit patients. ERJ Open Res.

[17] Hagens LA, Heijnen NFL, Smit MR (2021). Diagnosis of acute respiratory distress syndrome (DARTS) by bedside exhaled breath octane measurements in invasively ventilated patients: protocol of a multicentre observational cohort study. Ann Transl Med.

[18] Hagens LA, Van der Ven F, Heijnen NFL (2022). Improvement of an interobserver agreement of ARDS diagnosis by adding additional imaging and a confidence scale. Front Med.

[19] van Oort PMP, White IR, Ahmed W (2021). Detection and quantification of exhaled volatile organic compounds in mechanically ventilated patients—comparison of two sampling methods. Analyst.

[20] Riley RD, Ensor J, Snell KIE (2020). Calculating the sample size required for developing a clinical prediction model. BMJ.

[21] Pate A, Riley RD, Collins GS, van Smeden M, Van Calster B, Ensor J, Martin GP (2023). Minimum sample size for developing a multivariable prediction model using multinomial logistic regression. Stat Methods Med Res.

[22] Kuhn M (2008). Building predictive models in R using the caret package. J Stat Softw.

[23] Garge NR, Bobashev G, Eggleston B (2013). Random forest methodology for model-based recursive partitioning: the mobForest package for R. BMC Bioinformatics.

[24] Robin X, Turck N, Hainard A, Tiberti N, Lisacek F, Sanchez JC, Muller M (2011). pROC: an open-source package for R and S+ to analyze and compare ROC curves. BMC Bioinformatics.

[25] Bos LD, Schultz MJ, Sterk PJ (2014). Exhaled breath profiling for diagnosing acute respiratory distress syndrome. BMC Pulm Med.

[26] Zhou M, Sharma R, Zhu H (2019). Rapid breath analysis for acute respiratory distress syndrome diagnostics using a portable two-dimensional gas chromatography device. Anal Bioanal Chem.

[27] Ware LB, Koyama T, Zhao Z (2013). Biomarkers of lung epithelial injury and inflammation distinguish severe sepsis patients with acute respiratory distress syndrome. Crit Care.

[28] Leblanc D, Bouvet C, Degiovanni F (2014). Early lung ultrasonography predicts the occurrence of acute respiratory distress syndrome in blunt trauma patients. Intensive Care Med.

[29] Villar J, Herran-Monge R, Gonzalez-Higueras E (2021). Clinical and biological markers for predicting ARDS and outcome in septic patients. Sci Rep.

[30] Broeckaert F, Bernard A (2000). Clara cell secretory protein (CC16): characteristics and perspectives as lung peripheral biomarker. Clin Exp Allergy.

[31] Lin J, Zhang W, Wang L, Tian F (2018). Diagnostic and prognostic values of club cell protein 16 (CC16) in critical care patients with acute respiratory distress syndrome. J Clin Lab Anal.

[32] Bos LD, Sterk PJ, Schultz MJ (2013). Volatile metabolites of pathogens: a systematic review. PLoS Pathog.

[33] Sunesson A, Vaes W, Nilsson C, Blomquist G, Andersson B, Carlson R (1995). Identification of volatile metabolites from five fungal species cultivated on two media. Appl Environ Microbiol.

[34] Borjesson T, Stollman U, Schnurer J (1992). Volatile metabolites produced by six fungal species compared with other indicators of fungal growth on cereal grains. Appl Environ Microbiol.

[35] Micheluz A, Manente S, Rovea M, Slanzi D, Varese GC, Ravagnan G, Formenton G (2016). Detection of volatile metabolites of moulds isolated from a contaminated library. J Microbiol Methods.

[36] Trefz P, Koehler H, Klepik K, Moebius P, Reinhold P, Schubert JK, Miekisch W (2013). Volatile emissions from *Mycobacterium avium subsp*. paratuberculosis mirror bacterial growth and enable distinction of different strains. PLoS ONE.

[37] Barberis E, Amede E, Khoso S (2021). Metabolomics diagnosis of COVID-19 from exhaled breath condensate. Metabolites.

[38] Berna AZ, Akaho EH, Harris RM (2021). Reproducible breath metabolite changes in children with SARS-CoV-2 infection. ACS Infect Dis.

[39] Whiteson KL, Meinardi S, Lim YW (2014). Breath gas metabolites and bacterial metagenomes from cystic fibrosis airways indicate active pH neutral 2,3-butanedione fermentation. ISME J.

[40] Bos LD, Meinardi S, Blake D, Whiteson K (2016). Bacteria in the airways of patients with cystic fibrosis are genetically capable of producing VOCs in breath. J Breath Res.

[41] Sanchez JM, Sacks RD (2006). Development of a multibed sorption trap, comprehensive two-dimensional gas chromatography, and time-of-flight mass spectrometry system for the analysis of volatile organic compounds in human breath. Anal Chem.

[42] Van Berkel JJ, Dallinga JW, Moller GM, Godschalk RW, Moonen E, Wouters EF, Van Schooten FJ (2008). Development of accurate classification method based on the analysis of volatile organic compounds from human exhaled air. J Chromatogr B Analyt Technol Biomed Life Sci.

[43] Nathani N, Perkins GD, Tunnicliffe W, Murphy N, Manji M, Thickett DR (2008). Kerbs von Lungren 6 antigen is a marker of alveolar inflammation but not of infection in patients with acute respiratory distress syndrome. Crit Care.

[44] Belizario JE, Faintuch J, Malpartida MG (2020). Breath biopsy and discovery of exclusive volatile organic compounds for diagnosis of infectious diseases. Front Cell Infect Microbiol.

