# Supplementary material for: Breath metabolomics for diagnosis of acute respiratory distress syndrome
Source: Crit Care. 2024 Mar 23;28:96. doi: 10.1186/s13054-024-04882-7 (PMC10960461; doi:10.1186/s13054-024-04882-7)
Supplement: Supplementary file 1 — Additional file 1. Table S1. Confidence scores for meeting the Berlin Definition. Figure S1. Va riable importance in the predictive model. Figure S2. Comparison of the relative abundance of VOCs in different groups based on classification of ARDS of both centers. Figure S3. Diagnostic accuracy of the VOC-ARDS score classifier per center. Figure S4. Calibration plot of the prediction model. Figure S5. Diagnostic accuracy in the samples from the second measurement. Table S2. Characteristics of the patient cohort used to validate the VOC-ARDS score. Table S3. Development of the VOC-ARDS score. Table S4. Patient characteristics for AMC cohort. Table S5. Patient characteristics for MUMC cohort. Table S6. Biomarkers with abbreviations. Table S7. Spearman correlation coefficients between plasma KL-6 and the selected five VOCs. [file 13054_2024_4882_MOESM1_ESM.docx]

**Online supplement**

**Breath metabolomics for diagnosis of acute respiratory distress syndrome**

Shiqi Zhang*^1^, Laura A. Hagens*^1^, Nanon F.L. Heijnen^1^, Marry R. Smit^1^, Paul Brinkman^2^, Dominic Fenn^2^, Tom van der Poll^2,3^, Marcus J. Schultz^1,4,5^, Dennis C.J.J Bergmans, Ronny M. Schnabel, Lieuwe D.J. Bos^1^, for the DARTS Consortium.

* These authors contributed equally, shared first authorship

**Content:**

Description of ARDS classification procedure and Table S1

Figures: S1 – S5

Tables: S2 – S7

Correspondence:

Shiqi Zhang, Amsterdam UMC, Intensive Care Medicine, Meibergdreef 9, Room G3-228, 1105 AZ Amsterdam, The Netherlands, Phone: +31 (0)6 5936 9304; Email: [s.zhang@amsterdamumc.nl](mailto:s.zhang@amsterdamumc.nl)

# Supplementary figures

## **Description of ARDS classification procedure**

The diagnosis of ARDS was based on the Berlin definition. For each patient, the clinical manifestations, chest imaging (CT and/or X-ray), ventilator setting and blood gas parameters were collected within 72h after intubation. Three experts scored the patient with 8-point scale (Table S1) according to what extent the Berlin criteria were met.

## **Table S1. Confidence scores for meeting the Berlin Definition.**

| **Score** | **1** | **2** | **3** | **4** | **5** | **6** | **7** | **8** |
| --- | --- | --- | --- | --- | --- | --- | --- | --- |
| **Confidence** | High | Moderate | Slight | Equivocal | | Slight | Moderate | High |
| **Diagnosis** | No ARDS | | | | ARDS | | | |

Collect all scores from each expert. First, confidence scores of a CT scan were evaluated to result in the ‘certain ARDS’ (when the average score of three experts >= 5 and the minimum score >= 3) or ‘certain no ARDS’ (when the average score of three experts <= 4 and the highest score <= 6) classification. If CT scan was not available or did not lead to a certain diagnosis, then confidence scores of the chest X-ray were used for ARDS classification. If confidence scores of the chest X-ray were inconclusive as well, then patients were classified as ‘uncertain ARDS’ diagnosis and were discussed by the experts in a consensus meeting where patients were classified either as ‘likely ARDS’ or ‘likely no ARDS’.

# **Supplementary figures**

## Figure S1. Variable importance in the predictive model.


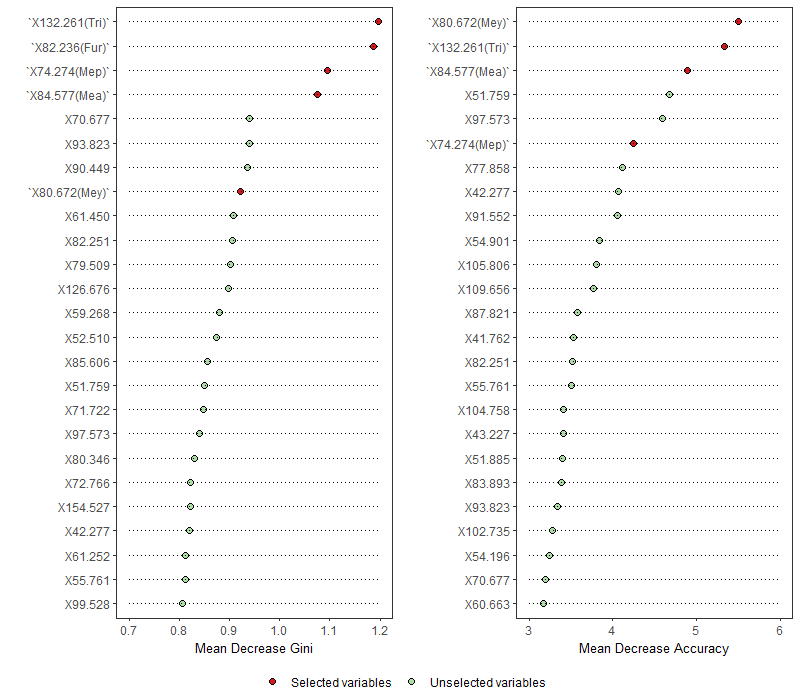


The variables on the y-axis represent different fragments of ionized volatile organic compounds (VOCs). The red dots indicate selected fragments, the green dots are unselected fragments. 2-methylfuran is not in the right panel due to its low ranking in Mean Decrease Accuracy. *Mey = 1-methylpyrrole; Tri = 1,3,5-trifluorobenzene; Mea = methoxyacetic acid; Fur = 2-methylfuran; Mep = 2-methyl-1-propanol.*

## **Figure S2.** Comparison of the relative abundance of VOCs in different groups based on classification of ARDS of both centers.


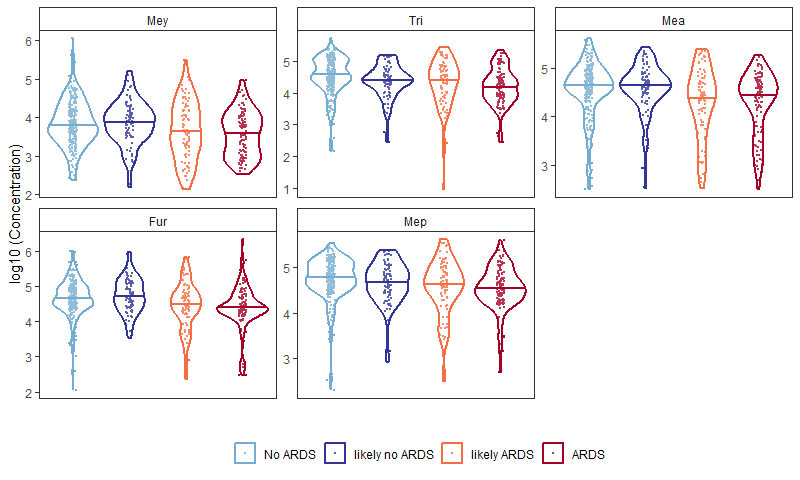


*VOCs=* *volatile organic compounds; Mey = 1-methylpyrrole; Tri = 1,3,5-trifluorobenzene; Mea = methoxyacetic acid; Fur = 2-methylfuran; Mep = 2-methyl-1-propanol.*

## **Figure S3.** Diagnostic accuracy of the VOC-ARDS score classifier per center.


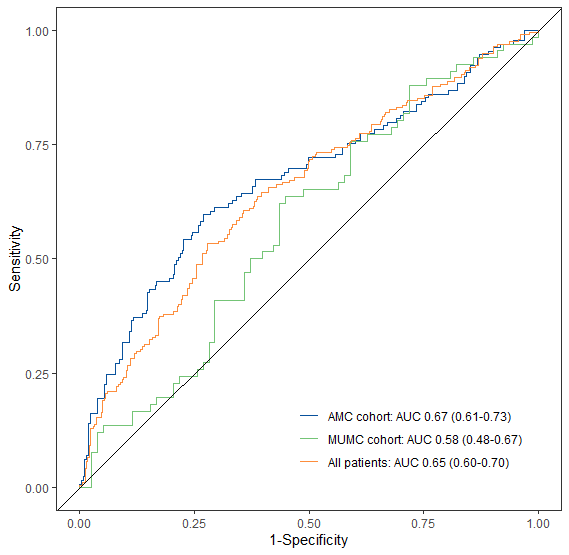


The blue line indicates the ROC curve in the AMC cohort (including patients with and without certain diagnosis), the green line indicates the ROC curve in the MUMC cohort (including patients with and without certain diagnosis), the orange line indicates the ROC curve of both centers combined, so the overall diagnostic accuracy in all included patients. *ROC curve= receiver operating characteristic curve; AUC = area under curve.*

## **Figure S4.** Calibration plot of the prediction model.


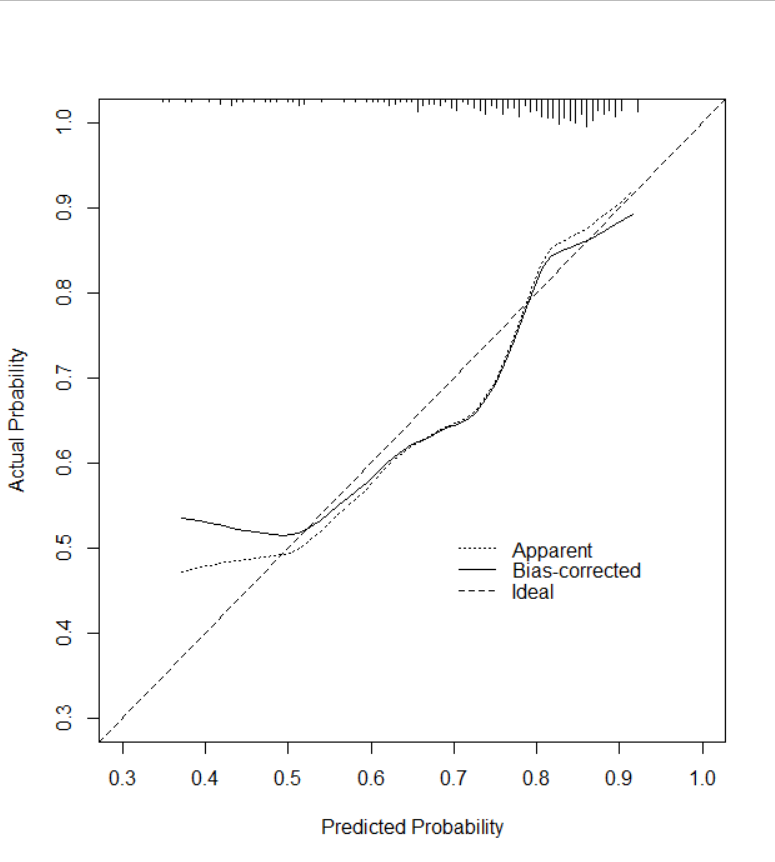


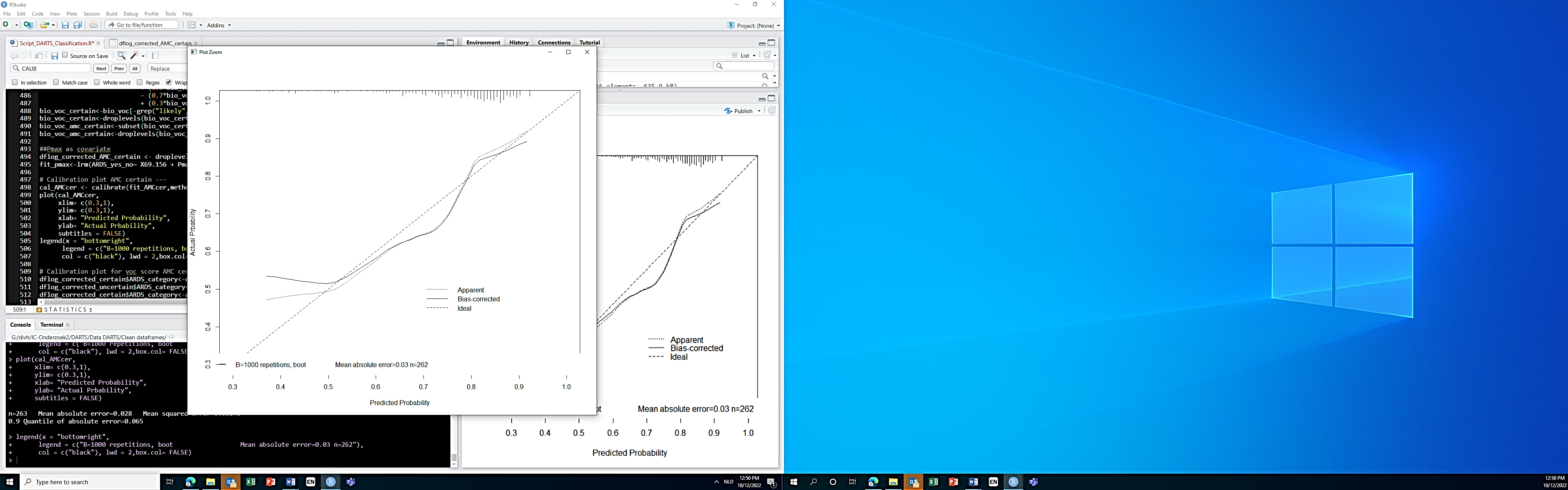


The calibration plot shows the coincidence between predicted probability and the actual probability of having ARDS.

## **Figure S5.** Diagnostic accuracy in the samples from the second measurement.


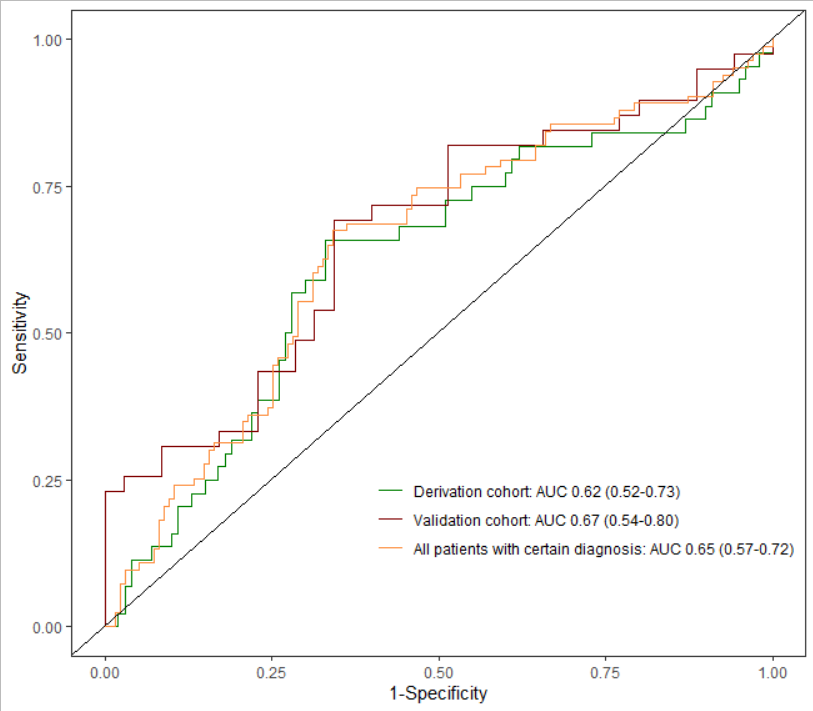


Sensitivity analysis was based on the second sample drawn the day after the first sample. The green line indicate ROC curve in the derivation cohort, the red line indicates the ROC curve in the validation cohort, the orange line indicates the ROC curve in all included patients with certain diagnosis. *ROC curve= receiver operating characteristic curve; AUC = area under curve.*

# Supplementary tables

## **Table S2.** Characteristics of the patient cohort used to validate the VOC-ARDS score.

|  | **Certain ARDS labels in MUMC** | |  |
| --- | --- | --- | --- |
|  | **ARDS**  **(n=47)** | **Non-ARDS**  **(n=52)** | **P value** |
| **Patients characteristics** | | | |
| **Age, years mean (SD)** | 63.6 (12.2) | 61.2 (16.2) | 0.399 |
| **Male, n (%)** | 34 (72.3) | 32 (61.5) | 0.355 |
| **Smoker, n (%)** | 33 (70.2) | 39 (75.0) | 0.758 |
| **BMI, kg/m2 median [IQR]** | 27 [24.1, 29.0] | 24.0 [22.6, 27.3] | 0.014 |
| **Admission characteristics** | | | |
| **Admission type, n (%)** | | | <0.001 |
| **Emergency surgical** | 1 (2.1) | 12 (23.1) |  |
| **Medical** | 46 (97.9) | 32 (61.5) |  |
| **Planned surgical** | 0 (0.0) | 8 (15.4) |  |
| **Admission condition, n (%)** |  |  |  |
| **Trauma** | 1 (2.1) | 15 (28.8) | 0.001 |
| **Neurosurgery** | 1 (2.1) | 6 (11.5) | 0.152 |
| **Shock** | 3 (6.4) | 8 (15.4) | 0.27 |
| **Extrapulmonary sepsis** | 3 (6.4) | 2 (3.8) | 0.908 |
| **Pancreatitis** | 1 (2.1) | 0 (0.0) | 0.959 |
| **Comorbidities, n (%)** |  |  |  |
| **Diabetes** | 10 (21.3) | 7 (13.5) | 0.446 |
| **Active malignancy** | 8 (17.0) | 5 (9.6) | 0.429 |
| **Immunocompromised** | 6 (12.8) | 3 (5.8) | 0.39 |
| **Cause of ARDS, n (%)** | | | <0.001 |
| **Non pulmonary** | 7 (14.9) | - |  |
| **Pulmonary** | 40 (85.1) | - |  |
| **ARDS severity, n (%)** | | | <0.001 |
| **Mild** | 2 (4.3) | - |  |
| **Moderate** | 27 (58.7) | - |  |
| **Severe** | 17 (37.0) | - |  |
| **Pneumonia, n (%)** | 35 (74.5) | 2 (3.8) | <0.001 |
| **Covid-19, n (%)** | 21 (44.7) | 0 (0.0) | <0.001 |
| **Apache II score, median [IQR]** | 17 [13, 24] | 24 [17, 26] | 0.067 |
| **SOFA score, median [IQR]** | 8 [6, 11] | 8 [5, 10] | 0.115 |
| **LIPS, median [IQR]** | 6 [5, 7] | 5 [4, 7] | 0.147 |
| **Pre ICU LOS, days median [IQR]** | 3 [2, 5] | 2 [1, 3] | 0.005 |
| **Mechanical ventilation and gas exchange, median [IQR]** | | |  |
| **P max, cmH2O** | 28 [25, 35] | 23 [19, 27] | 0.001 |
| **PEEP, cmH2O** | 11 [10, 12] | 8 [6, 8] | <0.001 |
| **Driving pressure, cmH2O** | 17 [15, 22] | 16 [12, 19] | 0.084 |
| **V_T_, mL** | 418 [369, 483] | 436 [357, 510] | 0.839 |
| **V_T_/PBW, ml/kg** | 6.4 [5.4, 7.4] | 6.8 [5.7, 7.8] | 0.464 |
| **Compliance,** **mL/cmH2O** | 23.9 [19.3, 30.6] | 25.03 [19.5, 32.9] | 0.655 |
| **RR, breaths/min** | 20 [18, 24] | 16 [15, 20] | 0.001 |
| **PaO_2_/FiO_2_, mmHg†** | 120 [79, 143] | 302 [184, 368] | <0.001 |
| **Duration MV, hours** | 24 [18, 33] | 24 [16, 33] | 0.741 |
| **Outcomes** | | | |
| **ICU LOS, days median [IQR]** | 11 [7, 24] | 6 [4, 14] | 0.01 |
| **Hospital LOS, days median [IQR]** | 27 [16, 40] | 16 [7, 24] | 0.003 |
| **ICU mortality, n (%)** | 19 (40.4) | 16 (30.8) | 0.428 |
| **30d mortality, n (%)** | 18 (38.3) | 19 (36.5) | 1 |
| **90d mortality, n (%)** | 23 (48.9) | 20 (38.5) | 0.397 |
| **1 year mortality, n (%)** | 25 (53.2) | 21 (40.4) | 0.283 |

Data are presented as n (%), median [IQR] or mean (SD). P-values were calculated using chi-square, T-test or Mann-Whitney U-test depending on the type and distribution of the variable. PBW is calculated as: PBW male = 50 + 0·91 * (cm of height – 152·4) and PBW female = 45·5 + 0·91 * (cm of height – 152·4).

† PF ratio worst measured in the 24 hours before sampling.

*ARDS = acute respiratory distress syndrome, BMI = body mass index, APACHE II = acute physiology and chronic health evaluation II, SOFA = sequential organ failure assessment, LIPS = lung injury prediction score, ICU = intensive care unit, LOS = length of stay, PaO2 = partial pressure of oxygen, FiO2 = fraction of inspired oxygen, MV = mechanical ventilation, PEEP = positive end-expiratory pressure, RR = respiratory rate. Vt = tidal volume, PBW = predicted body weight.*

## **TableS3.** Development of the VOC-ARDS score.

| **VOCs** | **Abbreviations** | **Regression coefficient** | **95CI** | |
| --- | --- | --- | --- | --- |
|  |  |  | **Lower limit** | **Upper limit** |
| 1-methylpyrrole | Mey | - 0.3 | -1.10 | 0.42 |
| 1,3,5-trifluorobenzene | Tri | 0.5 | -0.16 | 1.12 |
| Methoxyacetic acid | Mea | 0.7 | -0.10 | 1.41 |
| 2-methylfuran | Fur | 0.7 | 0.01 | 1.37 |
| 2-methyl-1-propanol | Mep | - 0.2 | -1.10 | 0.62 |

*VOCs = volatile compounds; ARDS = acute respiratory distress syndrome;* *Mey = 1-methylpyrrole; Tri = 1,3,5-trifluorobenzene; Mea = methoxyacetic acid; Fur = 2-methylfuran; Mep = 2-methyl-1-propanol.*

*The regression coefficients of the VOCs led to the VOC-ARDS score using the following formula:*

$VOC-ARDS score= -\log_{10} \left( Mey \right)\times0.3+\log_{10} \left( Tri \right)\times0.5+\log_{10} \left( Mea \right)\times0.7+\log_{10} \left( Fur \right)\times0.7-\log_{10} \left( Mep \right)\times0.2$

## **Table S4.** Patient characteristics for AMC cohort.

|  | **ARDS**  **(n=60)** | | **likely ARDS**  **(n=54)** | **likely no ARDS**  **(n=53)** | **No ARDS**  **(n=190)** |
| --- | --- | --- | --- | --- | --- |
| **Patients characteristics** | | | | | |
| **Age, years mean (SD)** | 60.2 (13.8) | | 63.5 (12.9) | 61.1 (13.7) | 61.8 (16.1) |
| **Male, n (%)** | 45 (75.0) | | 32 (59.3) | 40 (75.5) | 129 (68.3) |
| **Smoker, n (%)** | 33 (70.2) | | 16 (88.9) | 21 (84.0) | 39 (75.0) |
| **BMI, kg/m2 median [IQR]** | 26.9 [23.7, 30.7] | | 27.1 [24.9, 30.5] | 28.3 [24.8, 31.3] | 25.8 [23.0, 30.0] |
| **Admission characteristics** | | | | | |
| **Admission type, n (%)** | | | | | |
| **Emergency surgical** | 2 (3.3) | | 10 (18.5) | 7 (13.2) | 37 (19.6) |
| **Medical** | 54 (90.0) | | 39 (72.2) | 40 (75.5) | 122 (64.6) |
| **Planned surgical** | 4 (6.7) | | 5 (9.3) | 6 (11.3) | 30 (15.9) |
| **Admission condition, n (%)** |  | |  |  |  |
| **Trauma** | 2 (3.3) | | 5 (9.3) | 4 (7.5) | 34 (17.9) |
| **Neurosurgery** | 2 (3.3) | | 4 (7.4) | 12 (22.6) | 39 (20.5) |
| **Shock** | 1 (1.7) | | 5 (9.3) | 8 (15.1) | 15 (7.9) |
| **Extrapulmonary sepsis** | 3 (5.0) | | 11 (20.4) | 3 (5.7) | 31 (16.3) |
| **Pancreatitis** | 0 (0.0) | | 3 (5.6) | 0 (0.0) | 3 (1.6) |
| **Comorbidities, n (%)** |  | |  |  |  |
| **Diabetes** | 13 (21.7) | | 9 (16.7) | 9 (17.0) | 35 (18.4) |
| **Active malignancy** | 11 (18.3) | | 10 (18.5) | 8 (15.1) | 22 (11.6) |
| **Immunocompromised** | 5 (8.3) | | 3 (5.6) | 3 (5.7) | 10 (5.3) |
| **Cause of ARDS, n (%)** | | | | | |
| **Non pulmonary** | 4 (6.7) | | 21 (38.9) | - | - |
| **Pulmonary** | 56 (93.3) | | 33 (61.1) | - | - |
| **ARDS severity, n (%)** | | | | | |
| **Mild** | 3 (5.0) | | 9 (17.0) | - | - |
| **Moderate** | 30 (50) | | 26 (49.1) | - | - |
| **Severe** | 27 (45) | | 18 (34.0) | - | - |
| **Pneumonia, n (%)** | 54 (90.0) | | 29 (53.7) | 5 (9.4) | 27 (14.3) |
| **Covid-19, n (%)** | 29 (48.3) | | 9 (16.7) | 0 (0.0) | 1 (0.5) |
| **Apache II score, median [IQR]** | 20 [15, 22] | | 20 [18, 24] | 20 [15, 24] | 21 [15, 26] |
| **SOFA score, median [IQR]** | 8 [5, 12] | | 9 [7, 12] | 9 [8, 12] | 10 [8, 12] |
| **LIPS, median [IQR]** | 6 [6, 8] | | 6 [5, 8] | 4 [3, 7] | 5 [3, 6] |
| **Pre ICU LOS, days median [IQR]** | 2 [0, 5] | | 2.0 [1, 6] | 1 [0, 2] | 1 [0, 3] |
| **Mechanical ventilation and gas exchange, median [IQR]** | | | | | |
| **P max, cmH2O** | 23 [18, 28] | | 22.5 [17, 26] | 20 [16, 24] | 18 [14, 22] |
| **PEEP, cmH2O** | 10 [8, 12] | | 8 [7, 10] | 8 [6, 10] | 5 [5, 8] |
| **Driving pressure, cmH2O** | 12 [8, 17] | | 14 [9, 17] | 12 [9, 16] | 12 [8, 15] |
| **V_T_, mL** | 518 [386, 624] | | 483 [401, 561] | 463 [417, 612] | 468 [402, 532] |
| **V_T_/PBW, ml/kg** | 7.8 [6.3, 9.3] | | 7.4 [5.8, 8.8] | 7.1 [6.2, 8.3] | 7.1 [6.2, 8.6] |
| **Compliance,** **mL/cmH2O** | 38.2 [25.4, 61.9] | | 37.7 [26.1, 50.3] | 42.3 [29.8, 52.1] | 37.9 [27.8, 60.2] |
| **RR, breaths/min** | 21 [16, 28] | | 20 [15, 25] | 18 [15, 24] | 18 [15, 23] |
| **PaO_2_/FiO_2_, mmHg†** | 105 [81, 142] | | 119 [96, 180] | 174 [136, 228] | 261 [168, 336] |
| **Duration MV, hours** | 20 [10, 32] | | 22 [12, 26] | 20 [13, 26] | 22.0 [12, 32] |
| **Outcomes** | | |  |  |  |
| **ICU LOS, days median [IQR]** | | 9 [6, 19] | 7 [2, 12] | 5 [3, 9] | 6 [3, 11] |
| **Hospital LOS, days median [IQR]** | | 19 [10, 31] | 17 [12, 28] | 18 [9, 39] | 17 [8, 31] |
| **ICU mortality, n (%)** | | 25 (41.7) | 20 (37.0) | 14 (26.4) | 60 (31.6) |
| **30d mortality, n (%)** | | 25 (41.7) | 23 (42.6) | 19 (35.8) | 70 (36.8) |
| **90d mortality, n (%)** | | 27 (45.0) | 23 (42.6) | 21 (39.6) | 74 (38.9) |
| **1 year mortality, n (%)** | | 27 (45.0) | 24 (44.4) | 22 (41.5) | 76 (40.0) |

Data are presented as n (%), median [IQR] or mean (SD). P-values were calculated using chi-square, T-test or Mann-Whitney U-test depending on the type and distribution of the variable. PBW is calculated as: PBW male = 50 + 0·91 * (cm of height – 152·4) and PBW female = 45·5 + 0·91 * (cm of height – 152·4).

† PF ratio worst measured in the 24 hours before sampling.

*ARDS = acute respiratory distress syndrome, BMI = body mass index, APACHE II = acute physiology and chronic health evaluation II, SOFA = sequential organ failure assessment, LIPS = lung injury prediction score, ICU = intensive care unit, LOS = length of stay, PaO2 = partial pressure of oxygen, FiO2 = fraction of inspired oxygen, MV = mechanical ventilation, PEEP = positive end-expiratory pressure, RR = respiratory rate. Vt = tidal volume, PBW = predicted body weight.*

## **Table S5.** Patient characteristics for MUMC cohort.

|  | **ARDS**  **(n=47)** | **likely ARDS**  **(n=18)** | **likely no ARDS**  **(n=25)** | **No ARDS**  **(n=52)** |
| --- | --- | --- | --- | --- |
| **Patients characteristics** | | | | |
| **Age, years mean (SD)** | 63.6 (12.2) | 62.8 (12.7) | 61.2 (12.7) | 61.2 (16.2) |
| **Male, n (%)** | 34 (72.3) | 11 (61.1) | 14 (56.0) | 32 (61.5) |
| **Smoker, n (%)** | 33 (70.2) | 16 (88.9) | 21 (84.0) | 39 (75.0) |
| **BMI, kg/m2 median [IQR]** | 27.0 [24.1, 29.0] | 25.7 [23.6, 29.0] | 28.7 [24.5, 30.5] | 23.96 [22.6, 27.3] |
| **Admission characteristics** | | | | |
| **Admission type, n (%)** | | | | |
| **Emergency surgical** | 1 (2.1) | 2 (11.1) | 3 (12.0) | 12 (23.1) |
| **Medical** | 46 (97.9) | 12 (66.7) | 21 (84.0) | 32 (61.5) |
| **Planned surgical** | 0 (0.0) | 4 (22.2) | 1 (4.0) | 8 (15.4) |
| **Admission condition, n (%)** | | | | |
| **Trauma** | 1 (2.1) | 2 (11.1) | 3 (12.0) | 15 (28.8) |
| **Neurosurgery** | 1 (2.1) | 1 (5.6) | 2 (8.0) | 6 (11.5) |
| **Shock** | 3 (6.4) | 4 (22.2) | 3 (12.0) | 8 (15.4) |
| **Extrapulmonary sepsis** | 3 (6.4) | 4 (22.2) | 2 (8.0) | 2 (3.8) |
| **Pancreatitis** | 1 (2.1) | 0 (0.0) | 0 (0.0) | 0 (0.0) |
| **Comorbidities, n (%)** | | | | |
| **Diabetes** | 10 (21.3) | 4 (22.2) | 2 (8.0) | 7 (13.5) |
| **Active malignancy** | 8 (17.0) | 3 (16.7) | 0 (0.0) | 5 (9.6) |
| **Immunocompromised** | 6 (12.8) | 0 (0.0) | 1 (4.0) | 3 (5.8) |
| **Cause of ARDS, n (%)** | | | | |
| **Non pulmonary** | 7 (14.9) | 12 (66.7) | - | - |
| **Pulmonary** | 40 (85.1) | 6 (33.3) | - | - |
| **ARDS severity, n (%)** | | | | |
| **Mild** | 2 (4.3) | 8 (44.4) | - | - |
| **Moderate** | 27 (58.7) | 8 (44.4) | - | - |
| **Severe** | 17 (37.0) | 2 (11.1) | - | - |
| **Pneumonia, n (%)** | 35 (74.5) | 4 (22.2) | 2 (8.0) | 2 (3.8) |
| **Covid-19, n (%)** | 21 (44.7) | 2 (11.1) | 0 (0.0) | 0 (0.0) |
| **Apache II score, median [IQR]** | 19 (8) | 22 (8) | 21 (9) | 22 (8) |
| **SOFA score, median [IQR]** | 8 [6, 11] | 9 [6, 10] | 8 [7, 10] | 8 [5, 10] |
| **LIPS, median [IQR]** | 6 [5, 7] | 6 [5, 9] | 5 [4, 6] | 5 [4, 7] |
| **Pre ICU LOS, days median [IQR]** | 3 [2, 5] | 3 [1, 5] | 2 [1, 4] | 2 [1, 3] |
| **Mechanical ventilation and gas exchange, median [IQR]** | | | | |
| **P max, cmH2O** | 28 (8) | 27 (7) | 22 (6) | 23 (7) |
| **PEEP, cmH2O** | 11 [10, 12] | 8 [7, 12] | 8 [8, 8] | 8 [6, 8] |
| **Driving pressure, cmH2O** | 17 [15, 22] | 19 [14, 21] | 14 [10, 19] | 16 [12, 19] |
| **V_T_, mL** | 418 [369, 483] | 437 [390, 494] | 466 [430, 585] | 436 [357, 510] |
| **V_T_/PBW, ml/kg** | 6.43 [5.36, 7.35] | 7.14 [6.16, 7.63] | 7.57 [6.65, 8.57] | 6.76 [5.71, 7.84] |
| **Compliance,** **mL/cmH2O** | 24.0 [19.3, 30.7] | 24.4 [19.6, 34.4] | 32.8 [25.8, 54.0] | 26.9 [19.6, 36.5] |
| **RR, breaths/min** | 20 [18, 24] | 20 [17, 24] | 17 [13, 20] | 16 [15, 20] |
| **PaO_2_/FiO_2_, mmHg†** | 120 [79, 143] | 189 [119, 228] | 139 [119, 239] | 302 [184, 368] |
| **Duration MV, hours** | 24 [18, 33] | 20 [14, 26] | 24 [17, 30] | 24 [16, 33] |
| **Outcomes** | | | | |
| **ICU LOS, days median [IQR]** | 11 [7, 24] | 9 [4, 13] | 8 [5, 19] | 6 [4, 14] |
| **Hospital LOS, days median [IQR]** | 27 [16, 40] | 20 [7, 25] | 19 [12, 26] | 16 [7, 24] |
| **ICU mortality, n (%)** | 19 (40.4) | 5 (27.8) | 8 (32.0) | 16 (30.8) |
| **30d mortality, n (%)** | 18 (38.3) | 9 (50.0) | 10 (40.0) | 19 (36.5) |
| **90d mortality, n (%)** | 23 (48.9) | 11 (61.1) | 12 (48.0) | 20 (38.5) |
| **1 year mortality, n (%)** | 25 (53.2) | 11 (61.1) | 12 (48.0) | 21 (40.4) |

Data are presented as n (%), median [IQR] or mean (SD). P-values were calculated using chi-square, T-test or Mann-Whitney U-test depending on the type and distribution of the variable. PBW is calculated as: PBW male = 50 + 0·91 * (cm of height – 152·4) and PBW female = 45·5 + 0·91 * (cm of height – 152·4).

† PF ratio worst measured in the 24 hours before sampling.

*ARDS = acute respiratory distress syndrome, BMI = body mass index, APACHE II = acute physiology and chronic health evaluation II, SOFA = sequential organ failure assessment, LIPS = lung injury prediction score, ICU = intensive care unit, LOS = length of stay, PaO2 = partial pressure of oxygen, FiO2 = fraction of inspired oxygen, MV = mechanical ventilation, PEEP = positive end-expiratory pressure, RR = respiratory rate, Vt = tidal volume, PBW = predicted body weight.*

**Table S6.** Biomarkers with abbreviations.

| **Biomarker** | **Abbreviation** |
| --- | --- |
| Krebs von den Lungen-6 | KL-6 |
| Surfactant protein-D | SP-D |
| Club (Clara) cell protein 16 | CC-16 |
| Receptor for advanced glycation end-products | RAGE |
| Vascular endothelial growth factor | VEGF |
| Von Willebrand factor | vWF |
| Intercellular adhesion molecule | ICAM |
| Syndecan-1 protein | Syndecan-1 |
| Endothelial cell-specific molecule 1 | Endocan |
| E-selectin | E-selectin |
| Vascular adhesion molecule 1 | VCAM-1 |
| Angiopoietin-1 | *Ang-1* |
| Angiopoietin-2 | Ang-2 |
| The ratio of Angiopoietin-1/ Angiopoietin-2 | Ang2/1 |
| P-Selectin | P-selectin |
| Interferon γ | IFN- γ |
| Interleukin 6 | IL-6 |
| Interleukin 8 | IL-8 |
| Interleukin 10 | IL-10 |
| Interleukin-1 receptor antagonist | Interleukin 1RA |
| Interleukin 1β | IL-1β |
| Matrix metalloproteinase 8 | MMP-8 |
| Tumor-necrosis factor α | TNF-α |
| Tenascin C | TN-C |
| Granulocyte-macrophage colony-stimulating factor | GM-CSF |
| Pentraxin 3 | PTX3 |
| Tumor-necrosis factor receptor 1 | TNF R1 |
| Antithrombin | AT |
| D-dimer | D-dimer |
| Plasminogen activator inhibitor-1 | PAI-1 |
| Protein C | Protein C |
| FAS | FAS |
| Fas Ligand | Fas Ligand |

## **Table S7.** Spearman correlation coefficients between plasma KL-6 and the selected five VOCs.

| **VOCs** | **Correlation coefficients** | **95% CI** |
| --- | --- | --- |
| **Mey** | -0.130 | -0.238, -0.018 |
| **Tri** | -0.132 | -0.240, -0.020 |
| **Mea** | -0.103 | -0.212, 0 9 |
| **Fur** | -0.218 | -0.322, -0.108 |
| **Mep** | -0.170 | -0.277, -0.059 |

*VOCs = volatile organic compounds; Mey = 1-methylpyrrole; Tri = 1,3,5-frifluorobenzene; Mea = methoxyacetic acid; Fur = 2-methylfuran; Mep = 2-methyl-1-propanol; CI: Confidential Intervals.*
